# Publisher Correction: Dietary thiols accelerate aging of *C. elegans*

**DOI:** 10.1038/s41467-021-27126-6

**Published:** 2021-12-06

**Authors:** Ivan Gusarov, Ilya Shamovsky, Bibhusita Pani, Laurent Gautier, Svetlana Eremina, Olga Katkova-Zhukotskaya, Alexander Mironov, Alexander А. Makarov, Evgeny Nudler

**Affiliations:** 1 Department of Biochemistry and Molecular Pharmacology, New York University School of Medicine, New York, NY USA; 2 Engelhardt Institute of Molecular Biology, Russian Academy of Science, Moscow, Russia; 3 Howard Hughes Medical Institute, New York University School of Medicine, New York, NY USA

**Keywords:** Biochemistry, Ageing

Correction to: *Nature Communications* 10.1038/s41467-021-24634-3, published online 15 July 2021.

The original version of the Supplementary Information associated with this Article included an incorrect Supplementary Data 1 file, which was a copy of Supplementary Data 3 file. The correct Supplementary Data 1 file has now been added to the HTML version of the article.

## Supplementary information

Updated Supplementary Data 1

