# Supplementary material for: Dietary thiols accelerate aging of C. elegans
Source: Nat Commun. 2021 Jul 15;12:4336. doi: 10.1038/s41467-021-24634-3 (PMC8282788; doi:10.1038/s41467-021-24634-3)
Supplement: Supplementary file 1 — Supplementary Info [file 41467_2021_24634_MOESM1_ESM.docx]

**Supplementary Information**

**Supplementary Figure 1. Bacterial thiols and exogenously added NAC increase oxidative stress resistance and shorten*C. elegans* lifespan. a,** The increasing age of bacterial culture diminishes the negative effect of NAC on the lifespan. NGM plates with or without 5 mM NAC and seeded with *E. coli* OP50 were incubated for 2 days (compare to freshly seeded plates used in Fig. 1a) at 20^o^C prior to worm transfer (n= 228(untreated), 230(5mM NAC)). **b,** NAC slightly extends the lifespan in the presence of a high concentration of FUDR. NGM plates with 100 µM FUDR and +/-5 mM NAC were seeded with 10x *E. coli* OP50 and incubated for 1 hour at 20^o^C prior to worm transfer (n= 142(untreated), 141(5mM NAC)). Note that while 100 µM FUDR extends *C. elegans* lifespan, 40 µM FUDR does not (compare the untreated controls in Fig. 1c and e) and NAC has the opposite effect at these two concentrations. The medium percentage of lifespan change ±SD relative to an untreated control is indicated in red. **c,** Thiols supplemented exogenously promote oxidative stress resistance. N2 (wt) animals were grown on LB or LB+NAC plates. At day 2 of adulthood they were transferred to plates spotted with DB and supplemented with 150 mM paraquat. Error bars, mean ± SEM from 120 worms over 3 independent experiments. **d** and **e,** Diamide does not extend the lifespan of DB-fed worms. L4 stage worms were transferred on DB plates with or without 2.5 mM (n= 148(untreated), 190(diamide)), **d,** or 5 mM (n= 159(untreated), 149(diamide)), **e**, diamide. The medium percentage of lifespan change ±SD relative to an untreated control is indicated in red.

**Supplementary Figure 2. Phenotype enrichment analysis of genes up- or downregulated by NAC on DB.** For the analysis, we selected a list of genes which were statistically significantly (*q*<0.05) and affected by more than two-fold on DB+NAC compared to DB. The enrichment analysis tool is available on the Wormbase web site. *p*-values were calculated with the hypergeometric model tests and converted to *q*-values using a Benjamini-Hochberg step-up algorithm.

**Supplementary Figure 3. Analysis of *C. elegans* transcriptional response to NAC. a** and **b** Tables demonstrate the statistical significance of enriched terms among up- (**a**) and down- (**b**) regulated genes. NGR - number of annotated genes in the reference list; TNGR - total number of genes in the reference list; NG - number of annotated genes in the input list; TNG - total number of genes in the input list; Hyp - hypergeometric *p*-Value; Hyp* - corrected hypergeometric *p*-Value **c,** The most enriched gene ontology categories among genes upregulated by NAC. Analysis was performed by GeneCoDis3 program available online.

**Supplementary Figure 4. NAC downregulates genes involved in oxidative stress resistance. a,** Phenotype enrichment analysis of 272 genes downregulated by NAC treatment and upregulated by either SKN-1 or DAF-16 or both. The enrichment analysis tool is available on the Wormbase web site. *p*-values were calculated with the hypergeometric model tests and converted to *q*-values using a Benjamini-Hochberg step-up algorithm. Oxidative stress related categories are marked with arrows. **b** and **c,** A representative fluorescent image (**b**) and quantification (**c)** demonstrating that *skn-1*-dependent transcription is downregulated by thiols. L4 stage *gst-4::GFP* worms transferred to DB, DB+5 mM NAC or DB+5 mM GSH plates and incubated for two days prior imaging. Error bars, mean ± SEM from 30 worms over 3 independent experiments.

**Supplementary Figure 5. NAC suppresses SKN-1-dependent transcription to shorten *C. elegans* lifespan.** **a,** Genes oppositely regulated by NAC treatment and SKN-1 activation. Venn diagram demonstrating the overlap between genes downregulated by NAC and upregulated by the *wdr-23* deletion. RF=1.5, *p*-value= 2.07e-10. The analysis was performed by the online BioVenn program. **b,** Phenotype enrichment analysis of 229 genes downregulated by NAC and upregulated by the *wdr-23* deletion. Oxidative stress-related categories are marked with arrows. *p*-values were calculated with the hypergeometric model tests and converted to *q*-values using a Benjamini-Hochberg step-up algorithm. **c** and **d,** NAC does not shorten the lifespan of *wdr-23*-deficient worms. L4 stage *eri-1* worms were transferred on LB plates with (red line) or without (black line) 5 mM NAC and shifted to 25^o^C. Worms were reared on *E. coli* HT115 with empty vector (**c**) or *wdr-23* RNAi (**d)**. The mean percentage of lifespan change ±SD after NAC treatment relative to an untreated control is indicated in red (n= 307(e.v.), 318(e.v.+NAC), 272(*wdr-23*), 273(*wdr-23*+NAC). See also Supplementary Table 2.

**Supplementary Figure 6. Analysis of transcriptional response to DB diet. a** and **b,** Similarity of gene expression changes induced by DB diet and paraquat (PQ). Venn diagrams demonstrate the overlap among genes downregulated (RF= 4.5, *p*=0.000e+00) (**a**) and upregulated (RF= 1.7, *p*=1.224e-14) (**b)** by paraquat and DB. The list of paraquat-regulated genes was extracted from ref [22](#_ENREF_22). The analysis was performed by the online BioVenn program. **c** and **d,** Ontology enrichment analysis of genes similarly regulated by LB and DB+NAC compared to DB. **c,** Venn diagram demonstrating 355 commonly upregulated genes on LB and DB+NAC, and circular diagram showing the most enriched terms. **d,** Venn diagram demonstrating 124 commonly downregulated genes on LB and DB+NAC, and circular diagram showing the most enriched terms. The analysis was performed by online GeneCoDis3 and BioVenn programs. RF - representation factor is the number of overlapping genes divided by the expected number of overlapping genes and *p* is normal approximation of hypergeometric probability.

**Supplementary Figure 7. Regulation of endogenous GSH by acivicin. a,** Acivicin inhibits GSH import. WT worms were allowed to develop and grow at 20^o^C on NGM agar plates seeded with *E. coli* OP50. One-day old adults (~24 hours past L4 stage) were picked, washed 3 times in M9 buffer, and resuspended in M9 buffer (untreated), M9 with 5 mM GSH, or M9 with 5 mM GSH and 75 µM acivicin. After the incubation for 3 hours at 20^o^C with agitation, worms were washed 4 times with M9 buffer to remove extracellular chemicals followed by intracellular GSH measurement. Approximately, 200 worms were used for each experimental condition. The graph shows the mean ±SEM of three independent replicate experiments. *p*-values between untreated and GSH – 0.028 and GSH and GSH+acivicin – 0.04. **b**, Acivicin lowers the endogenous level of GSH only modestly. Worms were incubated on control and acivicin supplemented plates until day one of adulthood, picked, washed, and GSH concentration determined in lysates. Error bars, mean ± SEM from 3 independent experiments (n~600), two-tailed *t*-tests. **c,** γ-GCS inactivation by RNAi does not significantly upregulate the *tbb-6*::GFP expression. *tbb-6*::GFP expressing worms were incubated on *HT115* *E. coli* harboring either empty vector or vector expressing double strand RNAi against *gcs-1*. Box plots indicate median (middle line), 25th, 75th percentile (box) and 5th and 95th percentile (whiskers) as well as maximum, minimum and mean (single points). Total 55 worms were imaged in each group. *p*-value=0.43 (Student’s t-Test two-tailed distribution and two-sample equal variance). In all graphs *p*-values are: n.s. – not significant; *, p<0.05; **, p<0.01; ***, p < 0.001; ****, p < 0.0001; two-tailed t-tests.


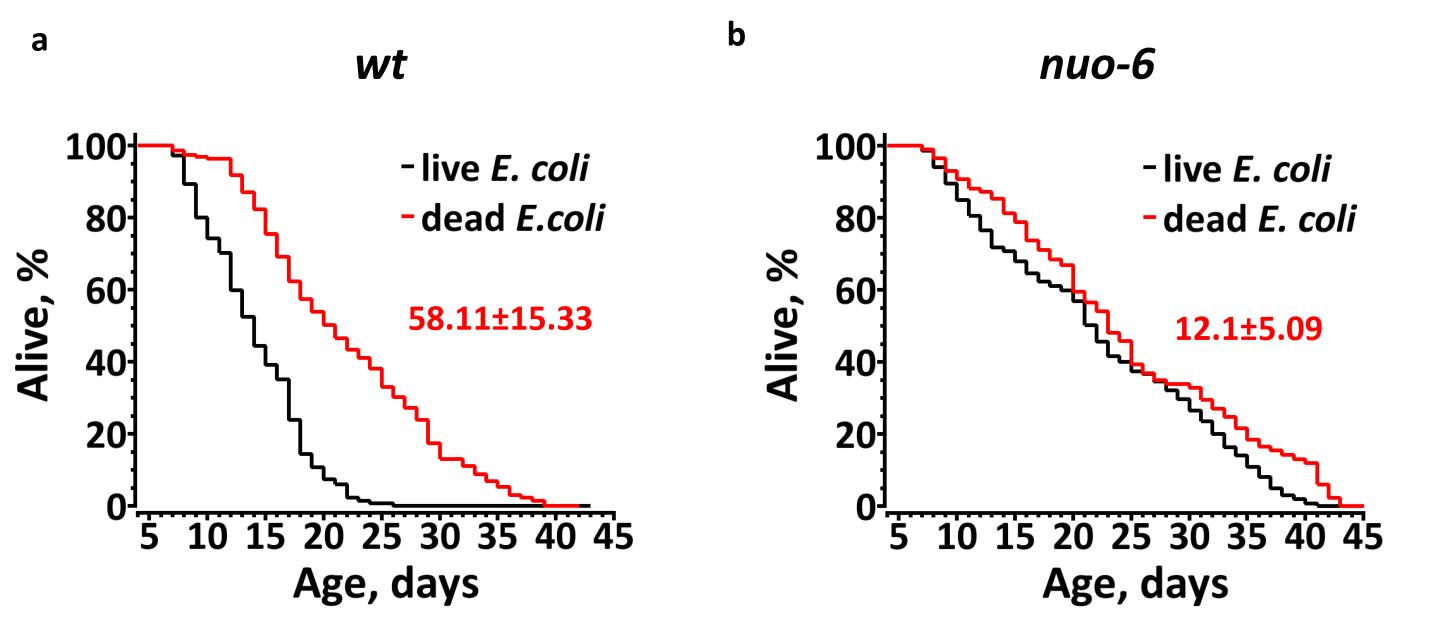
 **Supplementary Figure 8. A low thiol diet marginally increases the lifespan of *nuo-6* worms. a** and **b,** DB extends the lifespan of wt *C. elegans* but has a small effect on *nuo-6* mutants. L4 stage wt (**a**) or *nuo-6* (**b**) worms were transferred to LB (black curves) or DB (red curves) plates. The mean percentage change ±SD of worms reared on DB versus LB is indicated in red (n= 235(live, N2), 188(dead, N2), 127(live, *nuo-6*), 98 (dead, *nuo-6*)). See also Supplementary Table 2.

**Supplementary Figure 9. Acivicin does not upregulate the expression of chaperones. a** and **b,** A representative fluorescent image (**a**) and quantification (**b)** demonstrating the change of the expression of ER chaperone *hsp-4* by the increasing concentration of acivicin. **c** and **d,** A representative fluorescent image (**c**) and quantification (**d)** demonstrating the change of the expression of mitochondrial chaperone *hsp-6* by the increasing concentration of acivicin. n=25 worms per each conditions. Box plots indicate median (middle line), 25th, 75th percentile (box) and 5th and 95th percentile (whiskers) as well as maximum, minimum and mean (single points).

**Supplementary Figure 10. Interplay between acivicin and germline signaling. a,** If applied from eggs, acivicin delays development (left panel, n=223(untreated), 227(acivicin)) and decreases progeny production (right panel, n=18(untreated), 9(acivicin), *p*=0.0003). Error bars, mean ± SEM from 3 independent experiments. **b,** Acivicin shortens the long lifespan of *glp-1* worms. The mean percentage lifespan change ±SD of worms reared on acivicin versus untreated control is indicated in red (n= 216(untreated), 230(acivicin)). See also Supplementary Table 2. **c** and **d**, Germless worms contain a very low level of endogenous thiols, which cannot be further decreased by acivicin. Representative bright light (upper panel) and fluorescent (lower panel) images (**c**) and quantification (**d**) demonstrating total thiol staining in live wt and *glp-1* worms. Adult worms were maintained at 20^o^C and allowed to lay eggs on fresh plates without or with 75 µM acivicin. Worms allowed to develop and grow till stage L4 at 25^o^C. L4 stage worms transferred on fresh plates and incubated at 20^o^C. At day 2 of adulthood worms were stained with ThioFluor 623 (see Methods). n=53 worms per each conditions. Box plots indicate median (middle line), 25th, 75th percentile (box) and 5th and 95th percentile (whiskers) as well as maximum, minimum and mean (single points). In all graphs *p*-values are: n.s. – not significant; *, p<0.05; **, p<0.01; ***, p < 0.001; ****, p < 0.0001; two-tailed t-tests.

**Supplementary Figure 11. Effect of acivicin on human dermal fibroblasts. a,** Acivicin delays cell proliferation. Cells were treated with a range of acivicin concentrations as indicated, and the total number of viable cells were measured after 24 and 48 hours of incubation. **b**, Delay in cell growth is not due to apoptosis. Cells were treated with a range of acivicin concentrations as indicated, and apoptosis was measured by caspase 3/7 assay after 24 and 48 hours of incubation. **c** and **d**, Acivicin pretreatment increases cell resistance against tunicamycin (**c**) and heat (**d**) stresses. Cells were pretreated with a range of acivicin concentrations as indicated for 24 hours, washed, and subjected to 10 μg/ml tunicamycin for 48 hours (**c**) or lethal heat shock (45^o^C for 3 hours followed by 24 hours of recovery at 37^o^C) (**d**) followed by viable cell counting. **e**, Inactivation of Hsf1 does not abolish the protective effect of acivicin. Cells were treated with either non-target siRNA (NT) or siRNA targeting Hsf1 and then incubated with 10 µM acivicin for 24 hours. Cells were then subjected to 10 μg/ml tunicamycin for 48 hours followed by viable cell counting. Each graph shows the mean ±SEM cell counts from three independent experiments.

**Supplementary Tables**

**Supplementary Table 1. List of genes downregulated by NAC** **in the GO category: “Determination of adult lifespan”.**

| **Regulation by diet** | **Gene** | **Regulation** | **Proposed function^*^** |
| --- | --- | --- | --- |
| Down-regulated only by DB +NAC | *vha-6* | *skn-1* | vacuolar proton-translocating ATPase |
|  | *fat-7* | *skn-1* | delta-9 fatty acid desaturase |
|  | *ctl-3* | *daf-16* | catalase |
|  | *dod-22* | *daf-16* | No description available |
|  | *sid-2* |  | protein required for RNA interference |
|  | *elo-5* |  | polyunsaturated fatty acid (PUFA) elongase |
|  | *unc-52* |  | perlecan, heparan sulfate proteoglycan core protein |
|  | *vit-5* |  | vitellogenin, a lipid-binding protein precursor |
|  | *elo-2* |  | palmitic acid elongase |
|  | *cyp-34A9* | *daf-16* | cytochrome P450 family |
|  | *coq-1* |  | hexaprenyl pyrophosphate synthetase, ubiquinone biosynthesis |
|  | *let-805* |  | myotactin |
|  | *mig-6* |  | similar to tpapilin and lacunin |
|  | *npa-1* |  | precursor of fatty acids and retinol binding protein |
|  | *cdr-4* | *daf-16* | stress response to cadmium ion |
|  | *ifc-2* |  | intermediate filament protein |
|  | *ctl-2* | *daf-16* | catalase, required for normal lifespan of N2 and *daf-2* |
|  | *spp-12* | *daf-16* | SaPosin-like, defense response to Gram-positive bacterium |
|  | *dod-19* | *daf-16* | innate immune response |
|  | *gpd-3* | *daf-16* | glyceraldehyde 3-phosphate dehydrogenase |
|  | *ctl-1* | *daf-16* | catalase, contributes to the extended lifespan of *daf-2* |
|  | *ptr-23* |  | sterol sensing domain protein, required for normal molting |
|  | *ges-1* | *daf-16* | type B carboxylesterase |
|  | *pes-8* |  | required for regulation of adult lifespan |
|  | *mup-4* |  | required for junctional attachments between tissues |
|  | *ifb-1* |  | intermediate filament protein |
|  | *sodh-1* | *daf-16* | alcohol dehydrogenase , defense response |
|  | *adt-2* | *daf-16* | metallopeptidase |
|  | *fat-6* |  | delta-9 fatty acid desaturase |
|  | *cpr-1* |  | cathepsin B-like cysteine protease |
|  | *vit-4* |  | vitellogenin |
|  | *emb-9* |  | alpha-1 chain of Type IV basement membrane collagen |
|  | *vit-3* |  | vitellogenin |
|  | *lys-7* | *daf-16* | antimicrobial lysozyme |
|  | *acdh-1* | *mdt-15, daf2* | short-chain acyl-CoA dehydrogenase, fatty acid beta-oxidation |
|  | *stdh-1* | *daf-16* | steroid dehydrogenase required for normally short lifespan |
|  | *clec-186* |  | C-type lectin, innate immune response |
| Down-regulated by both DB+NAC and LB | *asp-3* |  | aspartyl protease |
|  | *mtl-1* | *daf-16* | metallothionein |
|  | *dod-3* | *daf-16* | No description available |
|  | *spp-1* | *daf-2* | caenopore |
|  | *sod-3* | *daf-16* | iron/manganese superoxide dismutase |
|  | *dod-24* | *skn-1, daf-16* | No description available |
|  | *nuc-1* |  | DNase II, which is required for DNA degradation |
|  | *asah-1* | *skn-1* | N-ACYLSPHINGOSINE AMIDOHYDROLASE |
|  | *asm-3* |  | No description available |
|  | *gst-10* | *skn-1* | glutathione S-transferase |
|  | *dod-6* | *daf-16* | No description available |

List of genes downregulated by dietary NAC in wt (N2) worms in the GO category – “Determination of adult lifespan” (See Fig. 2a and Supplementary Fig. 3). Worms were allowed to develop and grow on NGM agar plates with or without 15 mM NAC at 20^o^C until they reached day 8 of adulthood. Worms were collected and RNA was isolated and subjected to RNA-seq according to Illumina guidelines. Differentially expressed genes were determined as described in Material and Methods. ^*^-information from [www.wormbase.org](http://www.wormbase.org). The analysis was performed by the online GeneCoDis3 program.

**Supplementary Table 2. Summary of all aging experiments.**

| Rep-eats | *C. elegans* strain | *E.coli* strain/ RNAi | Media/ treatment | Number of animals that died /total | 50% survival, days | Mean survival, days ±SD | P-value,  2, 1 | Incre-ase/dec-rease, % | Mean increase decrease ,% ±SD |
| --- | --- | --- | --- | --- | --- | --- | --- | --- | --- |
|  |  |  |  |  |  |  |  |  |  |
| 1 | N2 | fresh^*^ OP50 | NGM | 68/94 | 16.44 | 17.02±0.84 |  |  |  |
| 2 |  |  |  | 73/86 | 16.63 |  |  |  |  |
| 3 |  |  |  | 93/107 | 17.98 |  |  |  |  |
| 1 |  | fresh^*^ OP50 | NGM  + 5 mM NAC |  |  | 15.44±0.57 | 0.00389* |  | -10.73 ±1.65 |
| 2 |  |  |  | 87/96 | 15.04 |  |  | -9.56 |  |
| 3 |  |  |  | 72/100 | 15.84 |  |  | -11.9 |  |
| 1 |  | fresh^*^ OP50 | NGM  + 15 mM NAC | 64/89 | 12.72 | 12.37±0.3 | 0.0169 | -22.63 | -27.15 ±4.84 |
| 2 |  |  |  | 87/96 | 12.21 |  |  | -26.58 |  |
| 3 |  |  |  | 72/100 | 12.18 |  |  | -32.26 |  |
|  |  |  |  |  |  |  |  |  |  |
| 1 | N2 | fresh^*^ OP50 | NGM | 68/94 | 13.4 | 14.15±0.66 |  |  |  |
| 2 |  |  |  | 73/86 | 14.61 |  |  |  |  |
| 3 |  |  |  | 93/107 | 14.45 |  |  |  |  |
| 1 |  | fresh^*^ OP50 | NGM  + 5 mM NAC | 64/89 | 12.14 | 12.88±0.93 | 0.00389* | -9.4 | -9.02 ±4.12 |
| 2 |  |  |  | 87/96 | 13.92 |  |  | -4.72 |  |
| 3 |  |  |  | 72/100 | 12.58 |  |  | -12.94 |  |
|  |  |  |  |  |  |  |  |  |  |
| 1 | N2 | 2d old^*^ OP50 | NGM | 57/75 | 15.34 | 17.34±1.74 |  |  |  |
| 2 |  |  |  | 88/100 | 18.51 |  |  |  |  |
| 3 |  |  |  | 83/114 | 18.16 |  |  |  |  |
| 1 |  | 2d old^*^ OP50 | NGM  +5 mM NAC | 51/75 | 14.86 | 17.08±2.05 | 0.5201 | -3.13 | -1.61 ±3.28 |
| 2 |  |  |  | 97/101 | 18.91 |  |  | 2.16 |  |
| 3 |  |  |  | 82/100 | 17.46 |  |  | -3.85 |  |
|  |  |  |  |  |  |  |  |  |  |
| 1 | N2 | OP50 | NGM | 85/100 | 14.5 | 14.58±0.11 |  |  |  |
| 2 |  |  |  | 90/100 | 14.65 |  |  |  |  |
| 3 |  |  |  |  |  |  |  |  |  |
| 1 |  | OP50 | NGM+5mM GSH | 82/100 | 13.03 | 12.55±0.69 | 0.17136 | -10.14 | -13.91± 5.33 |
| 2 |  |  |  | 86/100 | 12.06 |  |  | -17.68 |  |
| 3 |  |  |  |  |  |  |  |  |  |
|  |  |  |  |  |  |  |  |  |  |
| 1 | N2 | OP50 | NGM+Km+Cb | 47/60 | 18.32 | 21.22±2.54 |  |  |  |
| 2 |  |  |  | 51/60 | 23.04 |  |  |  |  |
| 3 |  |  |  | 50/60 | 22.31 |  |  |  |  |
| 1 |  | OP50 | NGM+ Km+  Cb+2.5 mM Diamide | 44/60 | 20.67 | 20.62±2.16 | 0.79766 | 12.83 | -1.74 ±16.73 |
| 2 |  |  |  | 68/80 | 18.43 |  |  | -20.00 |  |
| 3 |  |  |  | 78/90 | 22.75 |  |  | 1.97 |  |
|  |  |  |  |  |  |  |  |  |  |
| 1 | N2 | OP50 | NGM+ Km+Cb | 54/60 | 19.36 | 21.3±2.74 |  |  |  |
| 2 |  |  |  | 105/120 | 23.24 |  |  |  |  |
| 3 |  |  |  |  |  |  |  |  |  |
| 1 |  | OP50 | NGM+Km+Cb+ 5 mM Diamide | 45/60 | 14.44 | 16.01±2.22 | 0.04446 | -25.41 | -24.88 ±0.75 |
| 2 |  |  |  | 104/120 | 17.58 |  |  | -24.35 |  |
| 3 |  |  |  |  |  |  |  |  |  |
|  |  |  |  |  |  |  |  |  |  |
| 1 | *skn-1* (zu67) | OP50 | NGM | 44/50 | 11.2 | 11.78±0.62 |  |  |  |
| 2 |  |  |  | 76/80 | 11.7 |  |  |  |  |
| 3 |  |  |  | 68/72 | 12.44 |  |  |  |  |
| 1 |  |  | NGM+  5 mM NAC | 47/50 | 11.42 | 11.64±0.21 | 0.70158 | 1.96 | -1.06 ±4.59 |
| 2 |  |  |  | 63/70 | 11.84 |  |  | 1.20 |  |
| 3 |  |  |  | 67/70 | 11.65 |  |  | -6.35 |  |
|  |  |  |  |  |  |  |  |  |  |
| 1 | *daf-16*  mu86 | OP50 | NGM | 82/100 | 15.08 | 15.16±0.12 |  |  |  |
| 2 |  |  |  | 115/120 | 15.09 |  |  |  |  |
| 3 |  |  |  | 107/115 | 15.3 |  |  |  |  |
| 1 |  |  | NGM+  5 mM NAC | 80/100 | 11.48 | 12.19±0.61 | 0.01139 | -23.87 | -19.06 ±3.73 |
| 2 |  |  |  | 118/125 | 12.53 |  |  | -17.00 |  |
| 3 |  |  |  | 107/125 | 12.55 |  |  | -17.97 |  |
|  |  |  |  |  |  |  |  |  |  |
| 1 | N2, 25^o^C | HT115*+* pL4440 RNAi | NGM | 112/20 | 12.62 | 12.15±0.47 |  |  |  |
| 2 |  |  |  | 91/100 | 11.69 |  |  |  |  |
| 3 |  |  |  | 104/115 | 12.13 |  |  |  |  |
| 1 |  |  | NGM+ 5mM NAC | 110/120 | 11.2 | 10.64±0.51 | 0.022965 | -11.25 | -12.36 ±3.22 |
| 2 |  |  |  | 101/105 | 10.54 |  |  | -9.84 |  |
| 3 |  |  |  | 107/115 | 10.19 |  |  | -15.99 |  |
|  |  |  |  |  |  |  |  |  |  |
| 1 |  | HT115*+wdr-23* RNAi | NGM | 90/110 | 11 | 10.15±0.74 |  |  |  |
| 2 |  |  |  | 78/100 | 9.76 |  |  |  |  |
| 3 |  |  |  | 104/115 | 9.69 |  |  |  |  |
| 1 |  |  | NGM+5mM NAC | 93/110 | 11.53 | 10.88±0.58 | 0.02771 | 4.82 | 7.33 ±2.51 |
| 2 |  |  |  | 94/100 | 10.72 |  |  | 9.84 |  |
| 3 |  |  |  | 86/100 | 10.4 |  |  | 7.32 |  |
|  |  |  |  |  |  |  |  | | |
| 1 | N2 | OP50 | NGM | 90/100 | 15.46 | 15.52±0.45 |  |  |  |
| 2 |  |  |  | 79/100 | 16 |  |  |  |  |
| 3 |  |  |  | 87/100 | 15.11 |  |  |  |  |
| 1 |  |  | NGM+75µM Acivicin (from L4) | 97/105 | 18.42 | 18.36±0.79 | 0.005183 | 19.15 | 18.27 ±1.84 |
| 2 |  |  |  | 92/100 | 19.12 |  |  | 19.5 |  |
| 3 |  |  |  | 75/80 | 17.55 |  |  | 16.15 |  |
|  |  |  |  |  |  |  |  |  |  |
| 1 | *skn-1* | OP50 | NGM | 67/80 | 12.64 | 12.86±0.31 |  |  |  |
| 2 |  |  |  | 56/80 | 13.08 |  |  |  |  |
| 1 |  |  | NGM+75µM Acivicin (from L4) | 75/80 | 14.94 | 14.88±0.09 | 0.08945 | 18.19 | 15.71 ±3.51 |
| 2 |  |  |  | 71/80 | 14.81 |  |  | 13.22 |  |
|  |  |  |  |  |  |  |  |  |  |
| 1 | N2 | OP50 | NGM | 53/70 | 16.07 | 15.83±0.56 |  |  |  |
| 2 |  |  |  | 62/80 | 15.19 |  |  |  |  |
| 3 |  |  |  | 83/100 | 16.23 |  |  |  |  |
| 1 |  |  | NGM+75µM Acivicin (from eggs) | 92/100 | 20.95 | 20.84±0.25 | 0.001276 | 30.37 | 31.7 ±3.14 |
| 2 |  |  |  | 112/120 | 20.55 |  |  | 35.29 |  |
| 3 |  |  |  | 105/120 | 21.01 |  |  | 29.45 |  |
|  |  |  |  |  |  |  |  |  |  |
| 1 | *daf-16* | OP50 | NGM | 108/125 | 14.4 | 14.07±0.37 |  |  |  |
| 2 |  |  |  | 115/127 | 14.13 |  |  |  |  |
| 3 |  |  |  | 101/120 | 13.67 |  |  |  |  |
| 1 |  |  | NGM+75µM Acivicin (from eggs) | 120/125 | 14.48 | 14.91±0.64 | 0.280166 | 0.56 | 6.12 ±7.32 |
| 2 |  |  |  | 108/115 | 14.61 |  |  | 3.4 |  |
| 3 |  |  |  | 93/120 | 15.64 |  |  | 14.41 |  |
|  |  |  |  |  |  |  |  | | |
| 1 | N2 | OP50 | NGM  +Km+Cb | 50/75 | 24.15 | 25.49±1.28 |  |  |  |
| 2 |  |  |  | 54/80 | 25.64 |  |  |  |  |
| 3 |  |  |  | 50/88 | 26.69 |  |  |  |  |
| 1 |  |  | NGM+Km  +Cb+75µM Acivicin | 52/75 | 24.88 | 25.08±0.5 | 0.65953 | 3.02 | -1.46 ±5.40 |
| 2 |  |  |  | 63/72 | 25.65 |  |  | 0.04 |  |
| 3 |  |  |  | 78/83 | 24.7 |  |  | -7.46 |  |
| 1 |  | OP50 | NGM  +Km+Cb+  5 mM GSH | 68/87 | 19.91 | 20.5±0.85 | 0.005999 | -17.56 | -19.56 ±2.01 |
| 2 |  |  |  | 69/90 | 20.11 |  |  | -21.57 |  |
| 3 |  |  |  | 54/80 | 21.47 |  |  | -19.56 |  |
| 1 |  |  | NGM+Km  +Cb+Acivicin+GSH | 77/94 | 26.23 | 25.00±1.08 | 0.75197 | 8.61 | -1.62 ±9.25 |
| 2 |  |  |  | 78/92 | 24.6 |  |  | -4.06 |  |
| 3 |  |  |  | 85/93 | 24.18 |  |  | -9.40 |  |
|  |  |  |  |  |  |  |  | | |
| 1 | N2 | Heat inacti-vated OP50 | NGM  +Km+Cb | 105/110 | 24.25 | 23.04±1.18 |  | | |
| 2 |  |  |  | 133/135 | 24.03 |  |  |  |  |
| 3 |  |  |  | 128/135 | 23.29 |  |  |  |  |
| 4 |  |  |  | 59/75 | 22.02 |  |  |  |  |
| 5 |  |  |  | 100/112 | 21.61 |  |  |  |  |
| 1 |  |  | NGM  +Km+Cb  +15mM NAC | 70/90 | 14.43 | 14.68±1.03 | 0.0086 | -40.49 | -38.39± 5.53 |
| 2 |  |  |  | 62/80 | 13.8 |  |  | -42.57 |  |
| 3 |  |  |  | 79/80 | 15.81 |  |  | -32.11 |  |
| 1 |  |  | NGM  +Km+Cb  +5mM NAC | 86/100 | 21.29 | 20.26±0.93 | 0.017632 | -12.21 | -10.4± 2.57 |
| 4 |  |  |  | 73/85 | 19.48 |  |  | -11.54 |  |
| 5 |  |  |  | 104/111 | 20 |  |  | -7.45 |  |
|  |  |  |  |  |  |  |  |  |  |
| 1 | N2 | Heat inacti-vated OP50 | NGM  +Km  +Cb | 87/90 | 20.05 | 20.34±1.25 |  |  |  |
| 2 |  |  |  | 95/100 | 21.7 |  |  |  |  |
| 3 |  |  |  | 79/80 | 19.26 |  |  |  |  |
| 1 |  |  | NGM+ Km+  Cb+5mM NAC@day13 | 107/110 | 19.15 | 19.02±1.35 | 0.02738 | -4.49 | -6.52 ±2.07 |
| 2 |  |  |  | 103/110 | 20.3 |  |  | -6.45 |  |
| 3 |  |  |  | 79/80 | 17.6 |  |  | -8.62 |  |
|  |  |  |  |  |  |  |  | | |
| 1 | N2 | OP50 | NGM  +Km+Cb | 72/80 | 24.63 | 23.38±1.77 |  | | |
| 2 |  |  |  | 57/75 | 24.15 |  |  |  |  |
| 3 |  |  |  | 74/85 | 21.36 |  |  |  |  |
| 1 |  |  | NGM  +Km+Cb  +5mM NAC | 68/75 | 18.91 | 19.85±1.3 | 0.03721 | -23.22 | -14.82± 7.35 |
| 2 |  |  |  | 67/75 | 21.34 |  |  | -11.64 |  |
| 3 |  |  |  | 69/80 | 19.31 |  |  | -9.6 |  |
| 1 |  |  | NGM  +Km+Cb  +5mM NAS | 71/85 | 21.14 | 23.21±2.19 | 0.92934 | -14.17 | 1.34± 11.12 |
| 4 |  |  |  | 66/80 | 25.5 |  |  | 5.59 |  |
| 5 |  |  |  | 84/95 | 23 |  |  | 4.55 |  |
|  |  |  |  |  |  |  |  |  |  |
| 1 | N2 | OP50 | NGM+100µM FUDR | 72/80 | 26.13 | 24.89±1.75 |  |  |  |
| 2 |  |  |  | 70/80 | 23.65 |  |  |  |  |
| 3 |  |  |  |  |  |  |  |  |  |
| 1 |  |  | NGM+100µM FUDR  + 5mM NAC | 72/80 | 27.25 | 26.7±0.78 | 0.23091 | 4.29 | 7.41±4.41 |
| 2 |  |  |  | 69/80 | 26.14 |  |  | 10.53 |  |
| 3 |  |  |  |  |  |  |  |  |  |
|  |  |  |  |  |  |  |  | | |
| 1 | *nuo-6* | OP50 | NGM | 58/99 | 20 | 21.15±1.63 |  |  |  |
| 2 |  |  |  | 69/101 | 22.3 |  |  |  |  |
| 3 |  |  |  |  |  |  |  |  |  |
| 1 |  |  | NGM  +Km+Cb | 51/101 | 21.7 | 23.75±2.9 | 0.21215 | 8.5 | 12.1±5.09 |
| 2 |  |  |  | 47/72 | 25.8 |  |  | 15.7 |  |
| 3 |  |  |  |  |  |  |  |  |  |
|  |  |  |  |  |  |  |  | | |
| 1 | N2 | OP50 | NGM | 72/100 | 13.37 | 13.28±1.44 |  |  |  |
| 2 |  |  |  | 68/100 | 14.67 |  |  |  |  |
| 3 |  |  |  | 85/100 | 11.8 |  |  |  |  |
| 1 |  |  | NGM  +Km+Cb | 53/75 | 22.4 | 20.88±1.41 | 0.01382 | 67.54 | 58.11±15.33 |
| 2 |  |  |  | 51/90 | 20.6 |  |  | 40.42 |  |
| 3 |  |  |  | 84/90 | 19.63 |  |  | 66.36 |  |
|  |  |  |  |  |  |  |  |  |  |
| 1 | *glp-1* | OP50 | NGM | 76/100 | 27.54 | 24.79±2.39 |  |  |  |
| 2 |  |  |  | 82/100 | 23.23 |  |  |  |  |
| 3 |  |  |  | 58/90 | 23.59 |  |  |  |  |
| 1 |  |  | NGM  +75µM Acivicin | 61/100 | 21.49 | 21.63±1.06 | 0.05234 | -21.97 | -12.17  ±9.95 |
| 2 |  |  |  | 85/100 | 22.75 |  |  | -2.07 |  |
| 3 |  |  |  | 84/100 | 20.65 |  |  | -12.46 |  |
|  |  |  |  |  |  |  |  |  |  |

Each data set (repeat) was fitted to a Boltzmann sigmoid curve and the mean survival time calculated. The % change in lifespan is expressed relative to the control in the same repeat experiment. Independent experimental and control analyses, which were performed side-by-side, are indicated by the same number (1, 2, or 3) in the first column. Increase (+) or decrease (-) in lifespan is indicated. *p*-values were calculated relative to the control animals in the same experiment using Student’s *t*-Test (two-tailed distribution and paired). *-p-value was calculated for all five replicates with 5 mM NAC.

**Supplementary Table 3. Thiols level in *C. elegans* depends on the diet.**

|  | Glutathione, nM/mg of protein | | | Total reduced thiols, nM/mg | | |
| --- | --- | --- | --- | --- | --- | --- |
| Diet  Experiment | Live *E. coli* | Dead  *E. coli* | Dead  *E. coli*  + 15 mM NAC | Live *E. coli* | Dead *E. coli* | Dead *E. coli*  + 15 mM NAC |
| 1 | 26.07 | 18.48 | 22.00 | 118.204 | 103.497 | 123.038 |
| 2 | 23.32 | 21.89 | 14.51 | 117.805 | 85.7826 | 109.424 |
| 3 | 23.94 | 14.53 | 18.90 | 137.953 | 122.357 | 125.098 |
| 4 | 27.68 | 20.23 | 16.56 | 146.59 | 113.613 | 112.811 |
| 5 | 21.26 | 11.99 | 14.60 | 128.706 | 108.064 | 123.38 |
| 6 | 26.95 | 15.48 | 20.52 | 93.824 | 84.864 | 100.497 |
| 7 | 17.37 | 20.56 | 25.29 | 107.804 | 82.6122 | 139.276 |
| 8 | 19.91 | 19.80 | 28.77 | 81.6689 | 75.4266 | 96.8176 |
| 9 | 25.16 | 20.68 | 32.71 |  |  |  |
| 10 | 22.46 | 21.44 | 31.48 |  |  |  |
| 11 | 22.025 | 10.18 | 14.18 |  |  |  |
| 12 | 34.82 | 16.79 | 14.79 |  |  |  |
| 13 | 19.68 | 19.77 | 20.99 |  |  |  |
|  |  |  |  |  |  |  |
| *p*-values | 0.003571988 | |  | 0.000870359 | |  |
|  |  | 0.04695214 | |  | 0.016700556 | |

Animals were reared on live (LB) or dead (DB) *E. coli* OP50. L4 stage animals were transferred to the LB, DB or DB+ 15 mM NAC plates. 2 days old worms, picked, washed, lysed and intracellular glutathione and total reduced thiols were determined. *p*-values are calculated using Student’s t-Test assuming two-tailed distribution and paired samples.

**Supplementary Table 4. Thiol staining in live *C. elegans* by ThioFluor 623.**

|  | Mean fluorescence, AU | | | | |
| --- | --- | --- | --- | --- | --- |
| Diet | LB | | | DB | |
| Treatment | 15mM Diamide | 5mM Diamide | untreated | untreated | 15 mM NAC |
|  | 22 | 340 | 479 | 104 | 383 |
|  | 29 | 68 | 535 | 412 | 387 |
|  | 1 | 174 | 683 | 73 | 301 |
|  | 63 | 73 | 507 | 345 | 283 |
|  | 73 | 264 | 581 | 116 | 173 |
|  | 34 | 54 | 427 | 174 | 288 |
|  | 24 | 86 | 514 | 235 | 269 |
|  | 24 | 41 | 242 | 96 | 355 |
|  | 10 | 117 | 475 | - | - |
|  | 16 | 451 | 564 | - | - |
|  | 17 | 252 | 703 | - | - |
|  | 449 | 109 | 89 | 50 | 609 |
|  | 143 | 260 | 72 | 177 | 232 |
| Total number of worms | 128 | 127 | 126 | 94 | 97 |

L4 stage animals were transferred to LB or DB plates supplemented with chemicals as indicated, incubated for three days, picked, washed and intracellular thiols stained with ThioFluor 623. Worms were anesthetized in a drop of 2% sodium azide and images were captured immediately using a Zeiss AxioZoom v16 microscope. *p*-values (Student’s t-Test two-tailed distribution and two-sample equal variance) are: between untreated LB and DB – 0.001, between DB and DB+NAC - 0.012, between LB and LB+5mM diamide - 0.0003, between LB and LB+15mM diamide - 4.37E-06, and between LB+5mM diamide and DB – 0.968.

**Supplementary Table 5. Regulation of *gst-4::GFP* expression by diet and NAC**

|  | Fluorescence, AU | | | |
| --- | --- | --- | --- | --- |
| Diet | Live *E.coil* | Live *E.coil* +15mM NAC | Dead *E.coil* | Dead *E.coil* +15mM NAC |
|  | 90.4 | 91.4 | 204.2 | 120.7 |
|  | 115.1 | 117.7 | 213.4 | 104.4 |
|  | 304 | 150.9 | 270.1 | 163.1 |
|  | 122.2 | 97.3 | 231.4 | 136.5 |
|  | 123.2 | 200 | 210.9 | 96.5 |
|  | 236.1 | 151.7 | 322.2 | 85 |
|  | 299.3 | 132.5 | 309.9 | 77 |
|  | 293.3 | 140.4 | 345.9 | 35.5 |
|  | 244.6 | 109.5 | 344 | 55.7 |
|  | 319.1 | 156.9 | 316.3 | 68.5 |
|  | 295.8 | 165.8 | 327.6 |  |
|  | 300.9 | 186.3 | 346.5 | 126.2 |
|  | 273.7 | 165.2 | 355 | 75.9 |
|  |  |  |  |  |
| Total number of worms | 65 | 65 | 65 | 60 |

Worms let develop and grow on live or antibiotic killed *E. coli* OP50. L4 stage animals were transferred to the plates +/- 15 mM NAC. Five days old adults, picked, anesthetized in a drop of 2% sodium azide and images were captured immediately using a Zeiss AxioZoom v16 microscope. *p*-values (Student’s t-Test two-tailed distribution and two-sample equal variance) are: between live *E. coli* and live *E.coli* +15 mM NAC diets – 0.002, live *E. coli* and dead *E. coli* diets – 0.048, between dead *E. coli* and dead *E.coli* +15 mM NAC diets – 6.88x10^-10^.

**Supplementary Table 6. DHE ROS staining in *C. elegans* depends on diet**

| Groups Diet | Mean fluorescence, AU | | |
| --- | --- | --- | --- |
|  | LB | DB | DB+NAC |
| 1 | 164 | 666 | 629 |
| 2 | 39 | 526 | 551 |
| 3 | 169 | 337 | 308 |
| 4 | 167 | 329 | 309 |
| 5 | 115 | 277 | 252 |
| 6 | 24 | 194 | 216 |
| 7 | 59 | 221 | 198 |
| 8 | 24 | 214 | 208 |
| 9 | 273 | 564 | 458 |
| 10 | 184 | 455 | 370 |
| 11 | 251 | 537 | 392 |
| 12 | 296 | 517 | 479 |
| 13 | 380 | 537 | 515 |
| 14 | 192 | 624 | 603 |
| 15 | 336 | 721 | 616 |
| 16 | 156 | 630 | 573 |
| 17 | 111 | 418 | 253 |
| Total number of worms | 91 | 94 | 91 |

L4 stage animals were transferred to LB, DB or DB+NAC plates, incubated for three days, picked, washed and ROS stained with DHE. Worms were anesthetized in a drop of 2% sodium azide and images were captured immediately using a Zeiss AxioZoom v16 microscope. Five to ten worms were imaged in group. *p*-values (Student’s t-Test two-tailed distribution, paired) between LB and DB is 8.34e-08, and between DB and DB+NAC - 0.0018.

**Supplementary Table 7. Acivicin dose-dependently decreases thiol levels in *C. elegans***

|  | ThioFluor 623 mean fluorescence, AU | | | |
| --- | --- | --- | --- | --- |
| Treatment | untreated | 75 µM Acivicin | 150 µM Acivicin | 300 µM Acivicin |
|  | 193 | 70 | 80 | 15 |
|  | 194 | 206 | 44 | 68 |
|  | 156 | 215 | 99 | 90 |
|  | 346 | 335 | 37 | 27 |
|  | 474 | 115 | 14 | 5 |
|  | 99 | 97 | 11 | 7 |
|  | 212 | 53 | 30 | 11 |
|  | 224 | 182 | 7 | 55 |
|  | 548 | 312 | 117 | 72 |
|  | 306 | 108 | 74 | - |
|  | 410 | 174 | 144 | - |
|  | 271 | 311 | 64 | - |
|  | 508 | 273 | 138 | - |
|  | 472 | 238 | 169 | - |
|  | 471 | 220 | 200 | - |
| *p*-values |  | 0.006024166 | 1.90727e-06 | 8.24319e-06 |
| Total number of worms | 126 | 126 | 124 | 68 |

L4 stage animals were transferred to the plates with various concentration of acivicin, incubated for three days, picked, washed and intracellular thiols stained with ThioFluor 623. Worms were anesthetized in a drop of 2% sodium azide and images were captured immediately using a Zeiss AxioZoom v16 microscope. *p*-values are calculated using Student’s t-Test assuming two-tailed distribution and two-sample equal variance.

**Supplementary Table 8. Regulation of *tbb-6::GFP* expression by acivicin and NAC**

|  | Fluorescence, AU | | | | | |
| --- | --- | --- | --- | --- | --- | --- |
| Treatment | untreated | 5mM NAC | 15mM NAC | Acivicin | Acivicin  + 5 mM NAC | Acivicin  + 15 mM NAC |
|  | 17 | 29.1 |  | 46 | 63.9 |  |
|  | 13.4 | 28.4 |  | 125 | 79 |  |
|  | 43 | 21 |  | 186 | 226.8 |  |
|  | 24.7 | 26.1 |  | 263.5 | 167.6 |  |
|  | 26 | 17.4 |  | 209.2 | 71.6 |  |
|  | 32.6 | 25.1 |  | 194.9 | 84.4 |  |
|  | 24.8 | 24.4 |  | 173.7 | 123.8 |  |
|  | 22.9 | 13 |  | 133.6 | 67 |  |
|  | 12.5 |  | 34 | 65 |  | 34 |
|  | 16.1 |  | 24.4 | 105.6 |  | 37.3 |
|  | 13 |  | 22.2 | 40.2 |  | 37 |
|  | 34.2 |  | 41.9 | 173.7 | 134.1 | 110.9 |
|  | 36.9 |  | 28.2 | 161.4 | 148.8 | 92.8 |
|  | 51.4 |  | 57.6 | 223.6 | 184.9 | 127.3 |
| Total number of worms | 70 | 40 | 30 | 70 | 55 | 30 |

L4 stage animals were transferred to the plates supplemented with NAC, acivicin, or combination of them, as indicated, incubated for two days, picked, anesthetized in a drop of 2% sodium azide and images were captured immediately using a Zeiss AxioZoom v16 microscope. *p*-values (Student’s t-Test two-tailed distribution and two-sample equal variance) are: between untreated and 5 mM NAC – 0.48, untreated and 15 mM NAC – 0.18, between untreated and acivicin – 3.66E-07, between acivicin and acivicin+5 mM NAC- 0.29, between acivicin and acivicin+15 mM NAC- 0.0197.

**Supplementary Table 9. Regulation of *tbb-6::GFP* expression by thiol-modulating drugs**

|  | Fluorescence, AU | | | | | | | |
| --- | --- | --- | --- | --- | --- | --- | --- | --- |
| Treatment | Etanol | AAP | untre-ated | Acivicin | 100 µM DNP | untre-ated | 50 µM Cd^2+^ |  |
|  | 51 | 82 | 48 | 86.5 | 62 | 16.6 | 85.2 |  |
|  | 56.6 | 60.6 | 54.6 | 73 | 60 | 23.6 | 46.2 |  |
|  | 61.2 | 62 | 51 | 86 | 68 | 32.25 | 46.15 |  |
|  | - | 75 | 53 | 114 | 67 | 26.5 | 44.8 |  |
|  | 56 | 74 | 62 | 93 | 66 | 30 | 26.3 |  |
|  | 36 | 51 | 25 | 82 | 73 | 35.1 | 53.8 |  |
|  | 25 | 50 | 18 | 88 | 67 | 33.8 | 36.7 |  |
|  |  |  |  |  |  | 41.8 | 40.1 |  |
|  |  |  |  |  |  | 39.2 | 58.1 |  |
|  |  |  |  |  |  | 38.1 | 50 |  |
|  |  |  |  |  |  | 30 | 69.7 |  |
|  |  |  |  |  |  | 36.3 | 46.1 |  |
|  |  |  |  |  |  | 26.4 | 42.7 |  |
|  |  |  |  |  |  |  |  |  |
| Total number of worms | 30 | 35 | 35 | 35 | 35 | 65 | 65 |  |

L4 stage animals were transferred to the plates supplemented with additives as indicated, incubated for one day, picked, anesthetized in a drop of 2% sodium azide and images captured immediately using a Zeiss AxioZoom v16 microscope. *p*-values (Student’s t-Test two-tailed distribution and two-sample equal variance) are: between etanol and acetaminophen (AAP) – 0.038, between untreated and 75 µM acivicin – 0.0001, between untreated and 100 µM dinitrophenol (DNP) – 0.0055, between untreated and 50µM cadmium chloride - 0.0056.

**Supplementary Table 10. Thiol restriction elevates mitochondrial ROS in *C. elegans***

| Groups Diet | Fluorescence, AU | |
| --- | --- | --- |
|  | untreated | + 75 µM acivicin |
| 1 | 1201.8 | 1288 |
| 2 | 1159.5 | 1261.7 |
| 3 | 806.8 | 953.2 |
| 4 | 1242.8 | 1729.7 |
| 5 | 1054.4 | 1431.1 |
| 6 | 945.4 | 1566.2 |
| 7 | 1082.9 | 1752 |
| 8 | 1305 | 1877 |
| 9 | 1205 | 1515 |
| 10 | 1344.9 | 1796.2 |
| 11 | 922.2 | 1445.5 |
| 12 | 766 | 971 |
| *p*-value | 0.001415 | |
| Total number of worms | 79 | 79 |

Worms let to developed till stage L3 on control and acivicin supplemented plates and stained with MitoTracker CM-H_2_X (see Methods). Worms were anesthetized in a drop of levamisole and images were captured immediately using a Zeiss AxioZoom v16 microscope. Five to ten worms were imaged in group. *p*-values (Student’s t-Test two-tailed distribution and two-sample equal variance).

**Supplementary Table 11. Heat shock resistance is modulated by cellular thiols.**

|  | Survival, % | | | | | | | | |
| --- | --- | --- | --- | --- | --- | --- | --- | --- | --- |
| Treatment | untre-ated | Acivicin | untre-ated | Acivicin | untre-ated | Acivicin | live E.coli | dead E.coli | dead E.coli+ NAC |
| Time at 35^o^C | 2.5 | | 3.5 | | 4.5 | | 3.5 | | |
|  | 33.3 | 85 | 22.5 | 52.5 | 0 | 22.5 | 2.6 | 67.9 | 50 |
|  | 41 | 90 | 0 | 47.5 | 0 | 5 | 15 | 72.5 | 33.3 |
|  | 59 | 74.5 | 0 | 72.5 | 0 | 17.2 | 2.5 | 15.9 | 30 |
|  | 43.8 | 83.3 |  |  |  |  | 7.5 | 35 | 10 |
|  |  |  |  |  |  |  | 30 | 82.1 | 23.8 |
|  |  |  |  |  |  |  | 0 | 33.3 | 17.1 |
|  |  |  |  |  |  |  | 2.5 | 60 | 11.4 |
|  |  |  |  |  |  |  |  |  |  |
| p-value | 0.0008 | | 0.0095 | | 0.045 | | 0.00092 | |  |
|  |  |  |  |  |  |  |  | 0.024 | |
| Total number of worms | 150 | 150 | 120 | 120 | 109 | 109 | 278 | 264 | 201 |

Animals were reared on live (LB) or dead (DB) *E.coli* OP50. L4 stage animals were transferred to the LB, DB or DB+ 15 mM NAC plates. 2 days old worms transferred on LB or DB plates incubated at 35^o^C for indicated amount of time and shifted back to 20^o^C. Survival calculated 48 hours post heat shock treatment. *p*-values are Student’s t-Test two-tailed distribution and two-sample equal variance.

**Supplementary Table 12. *glp-1* worms contain low levels of endogenous thiols**

|  | Mean fluorescence, AU | | | |
| --- | --- | --- | --- | --- |
|  | wt | | *glp-1* | |
| Treatment | untreated | 75 µM Acivicin | untreated | 75 µM Acivicin |
|  | 127.2 | 35.1 | 3.7 | 5.4 |
|  | 55.7 | 41.6 | 2.6 | 3.9 |
|  | 45.5 | 24.9 | 4.1 | 0.5 |
|  | 69.4 | 53.3 | 2.5 | 1.1 |
|  | 95.7 | 45.6 | 4.9 | 0.2 |
|  | 98.4 | 26.8 | 0.6 | 10.3 |
|  | 77.5 | 53.7 | 2.6 | 1.2 |
|  | 85.3 | 29.2 | 3.5 | 0.9 |
|  | 49.3 | 70.7 | 1.9 | 0.9 |
|  |  |  |  |  |
| *p*-values | 0.002841697 | |  |  |
|  | 2.55889E-07 | | |  |
|  |  |  | 0.854189312 | |
| Total number of worms | 53 | 53 | 53 | 53 |

N2 and *glp-1* worms were grown at 20^o^C, adults transferred on fresh plates +/- 75 µM acivicin, allowed to lay eggs at 25^o^C overnight. Adults were removed and eggs allowed to hatch and develop at 25^o^C. L4 stage animals were transferred to the plates +/- 75 µM acivicin, incubated for two days at 20^o^C, picked, washed and intracellular thiols stained with ThioFluor 623. Worms were anesthetized in a drop of 2% sodium azide and images were captured immediately using a Zeiss AxioZoom v16 microscope. *p*-values are calculated using Student’s t-Test assuming two-tailed distribution and two-sample equal variance.
