# Supplementary material for: Dietary thiols accelerate aging of C. elegans
Source: Nat Commun. 2021 Jul 15;12:4336. doi: 10.1038/s41467-021-24634-3 (PMC8282788; doi:10.1038/s41467-021-24634-3)
Supplement: Supplementary file 3 — Supplementary Data legends [file 41467_2021_24634_MOESM3_ESM.docx]

**Supplementary Data File description:**

**Supplementary Data 1.**

**Tab 1 - LB vs DB**

**List of genes regulated by dead *E. coli OP50* diet**

List of genes statistically significantly regulated (q-value<0.05) in A8 wt (N2) worms reared on DB versus LB diet. p-values were calculated used Wald test and then adjusted for multiple testing using the procedure of Benjamini and Hochberg (padj).

List of differentially expressed genes in wt *C. elegans* reared on DB versus DB+NAC

**Tab 2 - DB vs DB+NAC**

**List of genes regulated by NAC on dead E. coli OP50 diet**

List of genes statistically significantly regulated (q-value<0.05) in A8 wt (N2) worms reared on DB versus DB+15 mM NAC diet. p-values were calculated used Wald test and then adjusted for multiple testing using the procedure of Benjamini and Hochberg (padj)

**Supplementary Data 2.**

**List of genes regulated by acivicin in *C. elegans*.**

List of genes statistically significantly regulated (q-value<0.05) by the treatment of wt (N2) worms with 75 µM acivicin for 24 hours. p-values were calculated used Wald test and then adjusted for multiple testing using the procedure of Benjamini and Hochberg (padj)

**Supplementary Data 3.**

**List of genes regulated by acivicin in human fibroblasts**

List of genes statistically significantly regulated (q-value<0.05) by the treatment of human fibroblasts with 75 µM acivicin for 24 hours. p-values were calculated using Wald test and then adjusted for multiple testing using the procedure of Benjamini and Hochberg (padj)
